# Measurement of skin and target dose in post-mastectomy radiotherapy using 4 and 6 MV photon beams

**DOI:** 10.1186/1748-717X-8-270

**Published:** 2013-11-16

**Authors:** Melanie Fischbach, Roger A Hälg, Matthias Hartmann, Jürgen Besserer, Günther Gruber, Uwe Schneider

**Affiliations:** 1 Medical Physics, Radiotherapy Hirslanden, Witellikerstrasse 40, Zürich, CH-8032, Switzerland; 2 Science Faculty, University of Zürich, Zürich, Switzerland

## Abstract

**Background:**

For patients with high risk breast cancer and mastectomy, radiotherapy is the treatment of choice to improve survival and local control. Target dose is mainly limited due to skin reactions. The feasibility of using 4 MV beams for chest wall treatment was studied and compared to standard 6 MV bolus radiotherapy.

**Methods:**

Post-mastectomy IMRT was planned on an Alderson-phantom using 4 and 6 MV photon beams without/with a 0.5 cm thick bolus. Dose was measured using TLDs placed at 8 locations in 1 and 3 mm depth to represent skin and superficial target dose, respectively.

**Results:**

4 MV and 6 MV beams with bolus perform equally regarding target coverage. The minimum and mean superficial target dose for the 6 MV and 4 MV were 93.0% and 94.7%, and 93.1% and 94.4%, respectively. Regarding skin dose the 4 MV photon beam was advantageous. The minimum and mean skin dose for the 6 MV and 4 MV was 76.7% and 81.6%, and 69.4% and 72.9%, respectively. The TPS was able to predict dose in the build-up region with a precision of around 5%.

**Conclusions:**

The use of 4 MV photon beams are a good alternative for treating the thoracic wall without the need to place a bolus on the patient. The main limitation of 4 MV beams is the limited dose rate.

## Background

For patients with high risk breast cancer and mastectomy, postoperative radiotherapy is the treatment of choice to improve local control and survival [1-3]. The 2005 Oxford meta-analysis shows that for node positive patients post-mastectomy radiotherapy (PMRT) decrease significantly the risk of chest wall recurrence from 21% to 7.8% and that this improvement is also correlated with an improvement of survival [3]. Thus, delivering adequate radiation doses to the chest wall is crucial to reduce the risk of treatment failure [4]. Keeping radiation-induced side effects to the skin as low as possible, while providing the intended dose to the chest wall remains a challenge [5,6].

Commonly post-mastectomy radiotherapy (PMRT) is administered using low energy photon beams (usually around 6 MV) in combination with boluses made from tissue-equivalent material. The bolus should be thick enough to provide an adequate dose build-up in the superficial chest wall, while keeping the dose to the skin as low as possible. Andic et al. [7] evaluated the optimal frequency for bolus PMRT and recommended for a typical treatment of 50 Gy in 25 fractions with 6 MV x-rays to use the bolus for 15 fractions only.

Another possibility to improve the chest wall dose distribution for PMRT would be using a photon energy lower than 6 MV x-rays.

The purpose of the present study is to calculate and measure skin and target dose distributions in an Alderson Rando phantom irradiated with 4 MV x-rays without bolus, and with 6 MV with and without bolus.

## Methods

### Target definition and treatment planning

**Figure 1:**
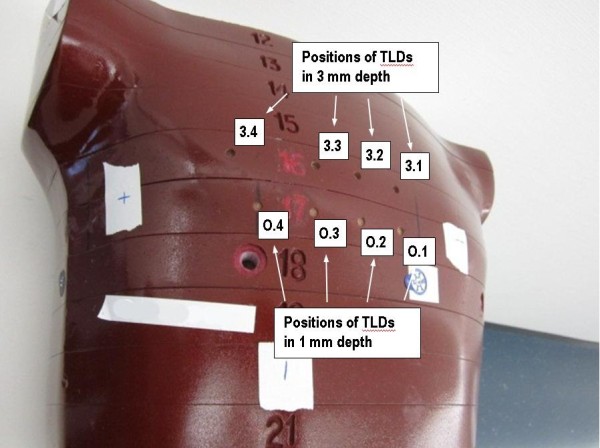
**Illustration of the Alderson phantom with the TLD measurement positions marked.** Please note that the ‘+’-marks are not relevant for this experiment.

An adult anthropomorphic Alderson-Rando phantom (RSD Radiology Support Devices, Long Beach, CA) was used consisting of 35 transversal slabs, made of material that was tissue equivalent for photon beams. A planning CT study of the phantom with 1.25 mm slice spacing was performed. The scan geometry is shown in Figure 1. The external surface of the patient and lung contours were defined with automated density gradient segmentation. The planning target volume of the chest wall was delineated according to following anatomical structures: Cranial: Caudal border of the clavicle head; Caudal: presumed inframammary fold; Anterior: 3 mm below the skin surface; Posterior: Rib-pleural interface; Lateral: Mid-axillary line; Medial: Sternal-rib junction. The distance of the PTV to the skin surface was chosen to be 3 mm since it was demonstrated that the blood vessels in the skin run within the first 5 mm under the epiderm (8. 9).

**Figure 2:**
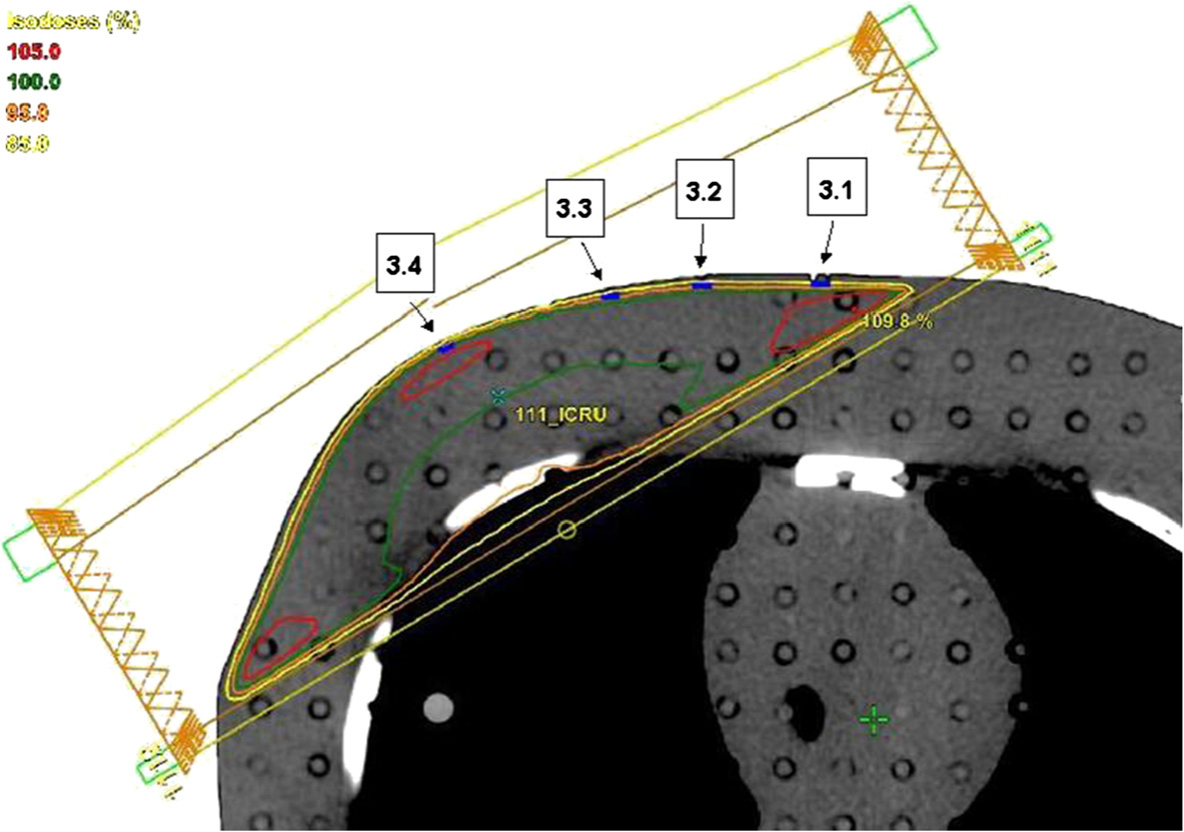
**Transversal slice at 0.1 cm cranial to the isocenter with the TLDs (in blue) in 3 mm depth.** For the 4 MV treatment plan the 85%, 95%, 100% and 105% isodoses are marked in yellow, orange, green and red, respectively. The geometry of the two tangential fields is shown.

We used for treatment planning the Eclipse External Beam Planning system version 10.0 (Varian Oncology Systems, Palo Alto, CA) using the analytic anisotropic algorithm (AAA, version 10.0.28). Treatment planning was performed according to the clinical standard at our institution with two isocentric tangential asymmetric treatment fields, both intensity modulated (IMRT) for virtual missing tissue compensation. The field geometry is shown in Figure 2. Field sizes were 7.7 cm and 14.5 cm in the cranial-caudal direction. Two photon beams with energies of 4 MV and 6 MV were used. The planned dose was 2 Gy per fraction with a total dose of 50 Gy. For the 6 MV beam two treatment plans were computed, one without bolus and one with 0.5 cm bolus material over the whole irradiation volume. According to the results of Andic et al. [7], which is the clinical practice in our institution, the 6 MV bolus treatment plan was weighted 60% and the non-bolus plan 40% for dose comparison. The treatment aim was a 95% coverage of the PTV. Treatment was administered using a Varian Truebeam linear accelerator with firmware 1.6 with sliding window technology (Varian Medical Systems, Palo Alto, CA, USA).

### Dose measurement

The dose measurements were performed using thermoluminescent dosimeters (TLDs) placed inside the Alderson-Rando phantom without casing (disks with a diameter of 4.50 mm and a thickness of 0.60 mm). The trading name of the detectors was TLD-100H (Thermo Fisher Scientific, Waltham, MA, USA). The useful dose range of the dosimeters was 1 μGy to 20 Gy according to the manufacturer.

TLDs were readout with a Fimel PCL3 isothermal TLD reader (PTW, Freiburg, Germany). A grey filter was applied during the detector read-out. Background dosimeters were used to correct the measurements for background signal. Reference dosimeters in each batch corrected for the daily variation in the TLD reader output. The dosimeters were calibrated using the 6 MV beam of a Varian Clinac 21 iX (Varian Medical Systems, Palo Alto, CA, USA) and a RW3 solid water slab phantom (PTW, Freiburg, Germany). For each TLD, a calibration factor in terms of dose per reader count was determined. The calibration was metrologically traceable to the Swiss National Metrology Institute (METAS). The reproducibility of a single dose measurement was 8% [8]. At each location measurements were repeated 9 to 11 times which resulted in a dose error between 2.4% and 2.7%.

A total of 8 measurement positions were selected in the phantom (Figure 1). A set of four 3 mm deep holes were drilled into the Alderson phantom in a plane 0.10 cm cranial to the isocenter. As the thickness of one TLD is 0.6 mm, the effective point of measurement for those TLDs was in 2.7 mm depth. Thus these measurement positions represented the superficial part of the PTV which is most critical regarding PTV-under-dosage and are marked as 3.1 – 3.4 in Figures 1 and 2. A second set of four holes each 1 mm in depth was drilled 2.65 cm caudal to the isocenter. The effective measuring depth of 0.7 mm represented the skin dose (marked as O.1 – O.4 in Figure 1). It should be noted that the holes and engraved numbers were filled with wax when the phantom was CT scanned to ensure precise dose calculation on the planning CT. On the planning CT the TLD positions were marked to obtain the computed dose at each measurement location. Before irradiation the phantom was positioned using two orthogonal kV set-up fields. Afterwards the TLDs were placed into the phantom and irradiated with one fraction (2 Gy) of the treatment plan. The measurements were repeated 9 to 11 times to reduce the measurement error. For the measurement, the 3 mm holes including the TLDs were closed with a plug made from Alderson soft tissue material. The TLDs in the 1 mm holes were irradiated open.

## Results

**Figure 3:**
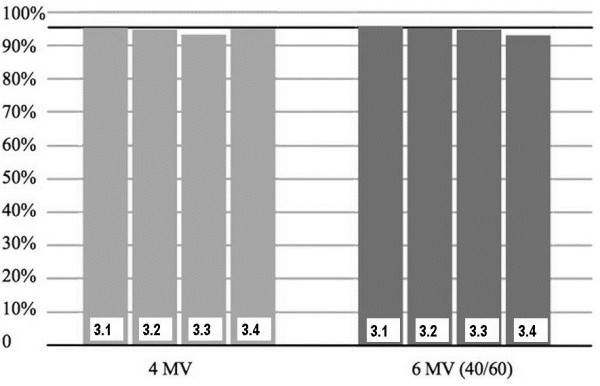
**Measured dose at the 4 locations in 3 mm depth as a percentage of the prescribed dose.** The light grey bars indicate the results from the 4 MV measurement and the dark grey bars from the 6 MV measurements without/with bolus using a 40/60 weighting.

The prescribed dose of a treatment fraction was 2 Gy. In Table 1 and Figure 3 the measured dose at the 4 locations in 3 mm depth is listed as a percentage of the prescribed dose. The light grey bars in Figure 3 indicate the results from the 4 MV measurement and the dark grey bars from the 6 MV measurements without/with bolus using a 40/60 weighting. In the figure the 95% dose is indicated by a solid line to show the treatment aim which was a 95% coverage of the PTV. The minimum and mean dose for the 6 MV treatment approach was 93.0% and 94.7%, respectively. For 4 MV the minimum and mean dose was 93.1% and 94.4%.

**Table 1:** Results of the TLD measurements at the four positions in 3 mm depth (3.1 – 3.4) and 1 mm depth (O.1 – O.4) in percentage of 2 Gy (prescribed dose per fraction) for the 4 MV and the 6 MV treatment plan

**Position**	**4 MV**	**6 MV (40/60)**
O.1	69.4	84.1
O.2	70.5	76.7
O.3	73.1	84.7
O.4	78.7	81.0
3.1	95.1	95.7
3.2	94.6	95.2
3.3	93.1	94.8
3.4	94.9	93.0

The 6 MV treatment without/with bolus was weighted 40/60.

**Figure 4:**
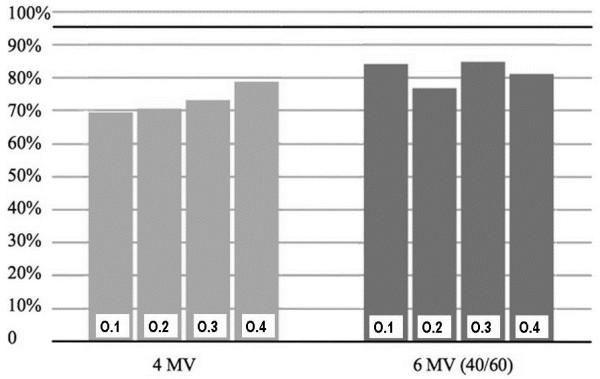
**Measured dose at the 4 locations in 1 mm depth as a percentage of the prescribed dose.** The light grey bars indicate the results from the 4 MV measurement and the dark grey bars from the 6 MV measurements without/with bolus using a 40/60 weighting.

The results of the measurements in 1 mm depth are listed in Table 1 and plotted in Figure 4. The minimum and mean dose for the 6 MV treatment approach was 76.7% and 81.6%, respectively. For 4 MV the minimum and mean dose was 69.4% and 72.9%.

In Table 2 the measured doses in Gy with the corresponding dose error (one standard deviation) is compared to the calculated dose values from Eclipse-AAA.

**Table 2:** Results of the TLD measurements and TPS calculations at the four positions in 3 mm depth (3.1 – 3.4) and 1 mm depth (O.1 – O.4) in Gy for the 4 MV and the 6 MV (without flab) treatment plan

**Position**	**4MV**			**6MV**		
	**Measured dose ± error/Gy**	**Computed dose/Gy**	**Difference/%**	**Measured dose ± error/Gy**	**Computed dose/Gy**	**Difference/%**
O.1	1.39 ± 0.03	1.41	1.4	1.23 ± 0.01	1.23	0.0
O.2	1.41 ± 0.03	1.38	-2.1	1.16 ± 0.01	1.25	7.8
O.3	1.46 ± 0.03	1.42	-2.7	1.35 ± 0.01	1.35	0.0
O.4	1.57 ± 0.02	1.55	-1.3	1.30 ± 0.02	1.37	5.4
3.1	1.90 ± 0.04	1.87	-1.6	1.73 ± 0.01	1.64	-5.2
3.2	1.89 ± 0.03	1.88	-0.5	1.78 ± 0.02	1.71	-3.9
3.3	1.86 ± 0.05	1.88	1.1	1.81 ± 0.02	1.74	-3.9
3.4	1.90 ± 0.06	1.95	2.6	1.74 ± 0.02	1.83	5.2

The 6 MV treatment without/with bolus was weighted 40/60. The last column indicates the difference between calculation and measurement.

## Discussion

PMRT decreases loco-regional recurrence and can increase survival in high-risk breast cancer patients [3]. The American Society of Clinical Oncology has published treatment guidelines, but has also indicated that the optimal technique for PMRT remains unknown.

Skin in itself may not be part of the clinical target volume (CTV) since recurrence on the skin scar is a rare event [9]. On the other hand the work from Van Limbergen in breast brachytherapy showed the importance avoiding the terminal branches of the skin microvessel that lay 3 mm below the skin surface [10]. So we excluded the first 3 mm of the skin surface from our CTV definition.

An underdosage of the most superficial part of the PTV is observed with 6 MV irradiation without bolus due to a larger build-up effect. This can be counterbalanced by an artificial tissue, so-called bolus. Its use enhances superficial dose, but also skin toxicity. On the other hand this might impact also local failure.

In 2004, an e-mail survey was sent to all active physician members of the American Society for Therapeutic Radiology and Oncology, the Canadian Association of Radiation Oncologists and the European Society for Therapeutic Radiology and Oncology. The survey focused on the technical details regarding the use of a bolus in PMRT. The results have been published recently [11]. In total, 1035 responses were obtained: 642 from the Americas (568 from the USA), 327 from Europe and 66 from Australasia. Respondents from the Americas were significantly more likely to always use a bolus (82%) than the Europeans (31%), as were the Australasians (65%) (P < 0.0001). The results also showed wide variation in the schedule of application (every day [33%] and alternate days [46%]) and thickness used (< 1 cm [35%] and > or = 1 cm [48%]). There is a wide variation in the use of a bolus in PMRT, and this probably translates into a variation in the dose delivered to the skin and may have an effect on local recurrence. No data are available about a possible impact of the use of a bolus and local failure.

The measurements performed in this work are an indicator that PMRT with 4 MV beams is equally good as the optimized [7] bolus/non-bolus (40/60) schedule with 6 MV photon beams when target coverage is important. With regard to skin sparing the 4 MV treatment is even advantageous reducing skin dose by about 10% of the prescribed dose. Another feasibility study was performed by Petoukhova et al. [12] for head and neck cancers. They found no significant difference between 4 MV and 6 MV radiation beams with regard to dose in and around air cavities.

The measured dose in the build-up region was also compared to the calculations of the Eclipse AAA algorithm. It was found that the precision of the computed dose is around 5%.

Taking the results of this work into account the use of 4 MV photon beams is advantageous to 6 MV with bolus. First, a dose distribution of the same quality can be administered to the PTV, while the use of 4 MV results in better skin sparing. Second, with 4 MV only one treatment plan must be computed instead of two with 6 MV, one without and one with bolus. Finally, no bolus must be placed on the patient which diminishes the potential of for getting the bolus for irradiation.

However, there are also disadvantages of using 4 MV beams for PMRT. In this work a Varian linear accelerator was used with limited dose-rate of 2.5 Gy/min at 4 MV. The dose-rate is 2.5 times lower than of the 6 MV beam resulting in longer irradiation times. This could be an additional problem when the deep-inspiration breath-hold technique, which is standard in our institution for left-sided breast cancer patients, is used. A further disadvantage can be the quality of the dose distribution of two tangential fields with large diameter targets. 4 MV photons may have worse quality of dose distribution in two tangential fields covering large diameter targets.

## Conclusions

Detailed skin and target dose measurements of 4 MV and 6 MV photon beams for post-mastectomy radiotherapy indicate that 4 MV beams are a good alternative for treating the thoracic wall without the need to place a bolus on the patient. Regarding skin dose the 4 MV beam performed even better than a 6 MV beam with 40/60 bolus schedule. It could be also shown that the treatment planning system Eclipse with the AAA algorithm was able to predict dose in the build-up region with a precision of around 5%. The clinical use of 4 MV beams to treat post-mastectomy patients is a question of technical issues. The main limitation is the limited dose rate which is at 4 MV 2.5 times smaller than at 6 MV.

## Competing interests

The authors declare that they have no competing interests.

## Authors’ contributions

MF performed this study as part of her Master thesis. RH assisted with the TLD measurements. MH helped with treatment planning. JB prepared the phantom and assisted with the dosimetry. GG was involved in the design of the study. US helped to perform the measurements and wrote the manuscript. All authors reviewed and approved the final manuscript.
